# Multifocal Electroretinogram Photopic Negative Response: A Reliable Paradigm to Detect Localized Retinal Ganglion Cells’ Impairment in Retrobulbar Optic Neuritis Due to Multiple Sclerosis as a Model of Retinal Neurodegeneration

**DOI:** 10.3390/diagnostics12051156

**Published:** 2022-05-06

**Authors:** Lucilla Barbano, Lucia Ziccardi, Giulio Antonelli, Carolina Gabri Nicoletti, Doriana Landi, Giorgia Mataluni, Benedetto Falsini, Girolama Alessandra Marfia, Diego Centonze, Vincenzo Parisi

**Affiliations:** 1 IRCCS—Fondazione Bietti, Via Livenza 1, 00198 Rome, Italy; lucilla.barbano@fondazionebietti.it (L.B.); giulio.antonelli@fondazionebietti.it (G.A.); vincenzo.parisi@fondazionebietti.it (V.P.); 2 Multiple Sclerosis Clinical and Research Unit, Department of Systems Medicine, Tor Vergata University, Via Montpellier 1, 00133 Rome, Italy; carolgabri@gmail.com (C.G.N.); doriana.landi@gmail.com (D.L.); giorgia.mataluni@gmail.com (G.M.); marfia@uniroma2.it (G.A.M.); centonze@uniroma2.it (D.C.); 3 Ophthalmology Department, IRCCS—Fondazione Policlinico Universitario A. Gemelli, Catholic University, Largo F. Vito 1, 00168 Rome, Italy; benedetto.falsini@unicatt.it; 4 Unit of Neurology and Neurorehabilitation, IRCCS—Neuromed, Via Atinense 18, 86077 Pozzilli, Italy

**Keywords:** multifocal electroretinogram, photopic negative response, retinal ganglion cells, multiple sclerosis, neurodegeneration

## Abstract

The measure of the full-field photopic negative response (ff-PhNR) of light-adapted full-field electroretinogram (ff-ERG) allows to evaluate the function of the innermost retinal layers (IRL) containing primarily retinal ganglion cells (RGCs) and other non-neuronal elements of the entire retina. The aim of this study was to acquire functional information of localized IRL by measuring the PhNR in response to multifocal stimuli (mfPhNR). In this case-control observational and retrospective study, we assessed mfPhNR responses from 25 healthy controls and from 20 patients with multiple sclerosis with previous history of optic neuritis (MS-ON), with full recovery of visual acuity, IRL morphological impairment, and absence of morpho-functional involvement of outer retinal layers (ORL). MfPhNR response amplitude densities (RADs) were measured from concentric rings (R) with increasing foveal eccentricity: 0–5° (R1), 5–10° (R2), 10–15° (R3), 15–20° (R4), and 20–25° (R5) from retinal sectors (superior-temporal (ST), superior-nasal (SN), inferior-nasal (IN), and inferior-temporal (IT)); between 5° and 20° and from retinal sectors (superior (S), temporal (T), inferior (I), and nasal (N)); and within 5° to 10° and within 10° and 20° from the fovea. The mfPhNR RAD values observed in all rings or sectors in MS-ON eyes were significantly reduced (*p* < 0.01) with respect to control ones. Our results suggest that mfPhNR recordings may detect localized IRL dysfunction in the pathologic condition of selective RGCs neurodegeneration.

## 1. Introduction

Several electrophysiological techniques have been developed for objective and selective clinical evaluation of the function of different retinal elements [1,2,3], such as photoreceptors and bipolar cells, which constitute the outer retinal layers (ORL), and retinal ganglion cells (RGCs) and their fibers, which constitute the innermost retinal layers (IRL)

The function of the ORL elements can be assessed by the full-field electroretinogram (ff-ERG) with non-structured light stimuli [2], whereas the pattern electroretinogram (PERG) recordings in response to contrast-reversing gratings or checkerboards [1,4,5,6,7,8,9,10] allows to evaluate the function of the IRL.

On the function of ORL, a non-structured light stimulus involves the whole retina contribution to the ff-ERG responses, and consequently, this method does not provide information on the function of cellular elements from localized retinal areas; this is because for a luminance stimulus presented to a localized retinal area, the obtained ERG responses reflect the bioelectrical activity of retinal elements located also outside of the stimulated retinal areas. This is due to the stray-light phenomenon that consists in an excitation of others retinal elements apart of those hit by the presented visual stimulus [11]. Thus, to obtain electrophysiological responses from localized retinal areas, it is necessary to minimize or abolish the stray-light effect [12] and to use adequate visual stimuli and rod-adapting background [13,14], both reducing this phenomenon. Based on adequate visual stimuli condition, and as they are able to minimize or abolish the stray-light effect, focal ERG (FERG) [12,15,16] and multifocal ERG (mfERG) [3,17] have been proposed as suitable methods to assess the bioelectrical activity from localized retinal areas and specifically from the macular region [3,18].

In the analysis of mfERG responses, by applying the “kernel analysis” with isolation of the first-order responses, it is possible to collect non-linear information of the ORL function [18]. Moreover, several mfERG studies, performed in retinal [19,20,21], systemic [22], or neurological disorders [23,24,25,26], suggested different modalities of analysis of the averaged responses to study selectively the ORL function, such as considering concentric annular retinal regions (the “ring analysis” [19,20,21,22,23,24,25,26] or different retinal sectors (the “sector analysis” [22,23,26])) centered on the fovea.

On the functional evaluation of the IRL, beside PERG recordings [9,10], the measure of the full-field photopic negative response (ff-PhNR) of light-adapted ff-ERG has been suggested [27,28,29,30,31,32,33,34].

The human ff-PhNR is a signal appearing as a slow, negative-going wave following the b-wave of the cone ERG and has been considered reliable to assess the function of the IRL [27,35]. More specifically, the neuronal origin of the ff-PhNR is from the IRL, containing RGCs and their fibers, with a potential contribution from non-neuronal elements, such as the Muller glial cells [36]. That the PhNR is originated by the above-mentioned retinal elements is supported by experimental studies performed in animal models [28,29]. In addition, since the ff-PhNR measurement is not influenced by several conditions (i.e., unknown refractive error, ocular media opacities, not stable target fixation during the recording session) [32,33,37,38], this method may present some advantages in the clinical practice with respect to PERG assessment that, instead, require the correction of refractive error, ocular media transparency, and stable target fixation for a reliable recordings.

As for ff-ERG, the ff-PhNR method assesses the RGCs bioelectrical activity of the whole retina and therefore cannot provide selective information on the function of the ganglionic elements of localized retinal areas.

In addition, as the focal and mfERG constitute an evolution of the ff-ERG for studying the function of localized ORL within the retinal arcades, it would be very useful to apply an electrophysiological method that allows the functional evaluation of localized IRL.

With this aim, Machida [34] reviewed the clinical applications of PhNR, suggesting the possibility to record responses from localized retinal areas by lowering the mfERG stimulus frequency and by using low-cut filters. Subsequently, his group [39] and Al-Nosairy [40] recorded the first-order kernel responses of PhNR obtained in response to multifocal visual stimuli (mfPhNR) to study the regional dysfunction of RGCs from five retinal areas in the glaucomatous optic neuropathy.

An intriguing goal for the functional evaluation of localized RGCs, similarly to that obtained with “ring” or “sector” analyses of mfERG [19,20,21,22,23,24,25,26] for the ORL function, can be to measure the PhNR responses by using appropriate visual stimuli to obtain bioelectrical responses from several localized (concentric or sectorial) retinal areas.

To understand whether the mfPhNR can assess localized RGCs dysfunction, it should be necessary to compare the bioelectrical responses obtained in normal subjects with those from patients presenting a morphological impairment of the IRL with a concomitant normal morpho-functional condition of the ORL. Accordingly, the study of patients with multiple sclerosis previously affected by retrobulbar optic neuropathy (MS-ON) with complete recovery of high-contrast visual acuity could be an adequate human model of retinal IRL neurodegeneration. This is based on the morpho-functional evidence (by Optical Coherence Tomography and by mfERG evaluations) of a reduction of volumes and thickness of IRL of the macular region (I-MV and I-MT) with a concomitant normal morphology and function of the macular ORL in MS-ON patients [26,41]. In addition, several studies showed that in MS-ON patients an IRL dysfunction (detectable by PERG recordings with reduced amplitudes) [42,43] and a normal or abnormal ORL function (detectable by recordings normal [44] or reduced [45] ff-ERG responses) may occur.

Therefore, the aim of our pivotal study was to evaluate whether the mfPhNR responses may provide functional information of ganglionic elements from localized retinal areas in MS-ON patients with a documented RGCs morphological involvement and a concomitant absence of morpho-functional changes of ORL.

Like the mfERG analysis [19,20,21,22,23,24,25,26], we measured mfPhNR amplitude responses from different retinal areas applying three retinal topographies for studying: (1) concentric annular rings centered to the fovea, (2) retinal sectors (superior-temporal, superior-nasal, inferior-temporal, and inferior-nasal with respect to the fovea), and (3) localized areas following the Early Treatment of Diabetic Retinopathy Study (ETDRS) [46] map configuration.

## 2. Materials and Methods

### 2.1. Study Design and Participants

All research procedures described in this work adhered to the tenets of Declaration of Helsinki. The study protocol (N.125/21/FB) was approved by the local Ethical Committee (Comitato Etico Centrale IRCCS Lazio, Sezione IFO/Fondazione Bietti, Rome, Italy), and upon recruitment, informed consent after full explanation of the procedure was obtained from each subject enrolled in the study.

Sixty-four relapsing, remitting MS patients were enrolled at the Visual Neurophysiology and Neurophthalmology Research Unit of IRCCS—Fondazione Bietti, referred by the Multiple Sclerosis center of the Tor Vergata University Hospital in Rome, between September 2021 and December 2021.

Based on our evidence [26] that MS-ON eyes with full recovery of high-contrast best corrected visual acuity (BCVA, 0.0 LogMAR) present absence of ORL pre-ganglionic elements dysfunction (normal mfERG responses) and a reduced I-MV and I-MT (suggesting morphological impairment of RGCs), we selected from the previously studied MS cohort [26] exclusively those MS-ON patients with normal mfERG responses and reduced I-MV and I-MT values.

The following demographic and clinical characteristics were adopted as inclusion criteria for the present study:

1. Age between 30 and 55 years;
2. Diagnosis of relapsing remitting MS according to validated 2010 McDonald criteria [47];
3. MS disease duration estimated as the number of years from onset to the most recent assessment of disability, ranging from 5 and 15 years;
4. Expanded Disability Status Scale, as ten-point disease severity derived from nine ratings for individual neurological domains [48], ranging from 0 to 3; this score was assessed by two trained [49] neurologists (D.L. and G.M.);
5. Treatment with disease-modifying therapies currently approved for preventing MS relapses, including Interferon-β-1a, Interferon-β-1b, Peginterferon beta-1a, Glatiramer acetate, Natalizumab, Dimethyl fumarate, and Teriflunomide [50];
6. A single episode of ON treated exclusively with steroid regimen following the Optic Neuritis Treatment Trial recommendations [51];
7. At least 12 months (ranging from 13 to 20 months) of time elapsed between the onset of ON and the inclusion in the study. This criterion was chosen since it is known that the retrograde degeneration following ON occurs over a period of 6 months [52]. When a MS patient was affected by ON in both eyes, we studied the eye affected longer that met the inclusion criteria;
8. Based on the ophthalmological examination, other inclusion criteria were: mean refractive error (when present) between −3.00 and +3.00 spherical equivalent; intraocular pressure less than 18 mmHg, absence of glaucoma, or other diseases involving cornea, lens (lens opacity classification system, LOCS III, stage < 1), uvea, retina;
9. High-contrast BCVA of 0.0 LogMAR of the ETDRS charts;
10. Absence of central scotoma or of square-wave jerks, saccadic intrusions, and nystagmus in primary position of gaze that can influence the ability to maintain a stable fixation during the mfPhNR recordings (see below);
11. Absence of other systemic diseases (i.e., diabetes, systemic hypertension, rheumatologic disorders) that may influence the retinal function.

Following these criteria, of the 64 enrolled patients, 20 MS patients (mean age 42.38 ± 5.28 years; 17 females and 3 males; mean MS disease duration 9.62 ± 5.36 years, range 5–20 years; mean Expanded Disability Status Scale score 1.49 ± 1.31, range 0–3) with previous history of a single unilateral or bilateral ON and with full recovery of high-contrast BCVA, providing 20 eyes, were selected and analyzed.

A group of selected 25 age-matched healthy subjects (mean age: 43.79 ± 6.11 years, 18 females and 7 males), providing 25 normal eyes, with BCVA of 0.0 LogMAR (mean 0.0 ± 0.0), served as controls.

### 2.2. Multifocal Photopic Negative Responses Recordings

The mfPhNR was recorded by using a modified version of Espion system (Diagnosys UK, Ltd.; Histon, Cambridge, UK).

**Figure 1:**
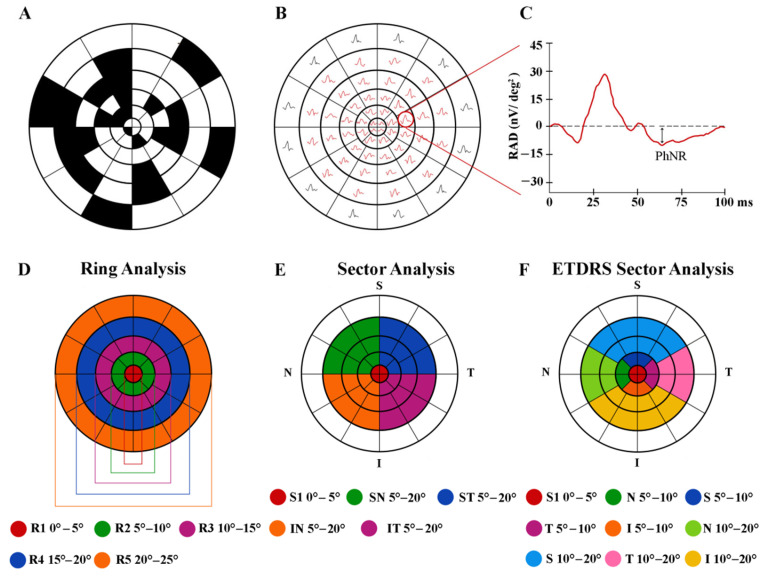
Multifocal photopic negative response (mfPhNR) stimulus (**A**) consisting of a circular dartboard of 60 elongated scaled elements and the relative obtained trace array (**B**) subtending 30° of the visual field from a representative control eye. The average response amplitude density (RAD) of the mfPhNR expressed in nanoVolt/degree^2^ (nV/deg^2^) (**C**) was measured as baseline-to-trough with an implicit time between 50 and 90 milliseconds (ms) from the stimulus onset. In the analysis of mfPhNR responses, we considered three different topographies. (**D**) The ring analysis analyzes mfPhNR RADs from five concentric annular areas (rings, R) with increasing eccentricity from the fovea, depicted in different colors: ring 1 (R1) in red from 0° to 5°; ring 2 (R2) in green 5° to 10°; ring 3 (R3) in purple from 10° to 15°; ring 4 (R4) in blue from 15° to 20°; and ring 5 (R5) in orange from 20° to 25°. (**E**) The sector analysis, covering an area of 20° of foveal eccentricity, analyzes RAD responses recorded from S1 (corresponding to R1, in red, see above), a circular area of 5° of centered on the fovea, and from the more external quarters of annulus within 5° and 20°, localized in the superior-temporal (ST, in blue), superior-nasal (SN, in green), inferior-nasal (IN, in orange), and inferior-temporal (IT, in purple) areas with respect to the fovea. (**F**) The ETDRS sector analysis analyzes the mfPhNR responses from nine sectors corresponding to the ETDRS map configuration. The central sector corresponds to the R1 of the ring analysis and to S1 of the sector analysis (in red, see above); the external sectors analyze the superior (S, in blue), temporal (T, in purple), inferior (I, in orange), and nasal (N, in dark green) areas within 5° and 10° from the fovea; and the outermost sectors analyze the S (in light blue), T (in pink), I (in yellow), and N (in light green) sectors within 10° and 20° from the fovea.

As reported in Figure 1A, the multifocal stimulus consists of a circular stimulus of 60 elongated scaled dart pattern “segments” presented in a monitor screen (size, 69 cm width and 38 cm height), with a mean background luminance of 200 cd/m^2^, at the viewing distance of 330 mm. Stimulus frequency was 7 Hz.

Each “segment” was independently alternated between black (0 cd/m^2^) and white (400 cd/m^2^) according to an m-sequence of 12 bits. Total recording time was average 20 min of several periods of about 30 s each. Between recording periods, the subject was allowed to rest for a few seconds.

The monitor screen presented a central fixation cross that was used as target, and each patient positively reported that he or she could clearly perceive the cross-fixation target. The eye’s position was monitored by a video system in the screen of the computer. In all subjects, mfPhNR was binocularly recorded in the presence of pupils that were maximally pharmacologically dilated with 1% tropicamide to a diameter of 7 to 8 mm. Pupil diameter was measured by an observer (G.A.) by means of a ruler and a magnifying lens and stored for each tested eye. The cornea was anesthetized with benoxinate 0.4% eye drops.

MfPhNR was recorded between an active Dawson Trick Litzkow bipolar contact electrode and a reference electrode (Ag/AgCl skin electrode placed on the corresponding outer canthi). A small Ag/AgCl skin ground electrode was placed in center of the forehead. Interelectrode resistance was lower than 3 KOhms. The signal was filtered (band pass 3–100 Hz) by Espion system. After automatic rejection of artifacts, the first-order kernel response was considered.

After the whole acquisition (Figure 1B), the obtained mfPhNR responses were analyzed. The average response amplitude densities (RAD) of the mfPhNR expressed in nanoVolt/degree^2^ (nV/deg^2^) were measured as baseline-to-trough (BT), that is, the difference between the pre-stimulus baseline and the more negative point in the trough with an implicit time between 50 and 90 ms from the stimulus onset (see Figure 1C).

The recorded mfPhNR responses were analyzed by using three different topographies centered on the fovea, as follows:

(1) Ring analysis: we used the same analysis proposed in other reports for mfERG responses [24,25,26]. This is made of five concentric annular areas (rings, R) with increasing eccentricity from the fovea: the first one analyzed a 5° radius circular area centered on the fovea (ring 1, R1), the second one analyzed the annular area enclosed between 5° and 10° (ring 2, R2), the third one analyzed the annular area enclosed between 10° and 15° (ring 3, R3), the fourth one analyzed the more external annular area between 15° and 20° (ring 4, R4), and the fifth one analyzed the outermost area between 20° and 25° (ring 5, R5) (Figure 1D).

(2) Sectors analysis: following previously described analyses for mfPhNR [34,39] responses, we analyzed five sectors covering an area of 20° of eccentricity from the fovea. The first sector (S1) corresponds to R1 (see above) analyzing responses from 5° radius area centered on the fovea; the more external sectors were quarters of annulus, localized in the superior-temporal (ST), superior-nasal (SN), inferior-nasal (IN), and inferior-temporal (IT) areas with respect to the fovea. The radius of the inner border of the annulus was 5°, and that of the outer border was 20° (Figure 1E).

(3) ETDRS sector analysis: following the Sd-OCT analysis of the macula [53], we studied the mfPhNR signals from localized areas corresponding to the ETDRS map configuration [46]. It consists of nine sectors, and the central one analyzes 5° radius circular area centered on the fovea, corresponding to the R1 of the ring analysis and to S1 of the sector analysis. Other external sectors analyze specifically the superior (S), temporal (T), inferior (I), and nasal (N) areas within 5° and 10° from the foveal center. The outermost sectors analyze S, T, I, and N areas within 10° and 20° from the foveal center (Figure 1F).

### 2.3. Statistical Analysis

We assumed a Gaussian distribution of our data. The normal distribution was assessed by using the Kolmogorov–Smirnov test. Size estimates were obtained from pilot evaluations performed in 10 eyes from 10 MS-ON eyes and 12 eyes from 12 control subjects other than those included in the current study (unpublished data).

Therefore, considering the mean R1 mfPhNR RAD value of descriptive statistic equal to 31.1 nV/deg^2^, standard deviation (SD) = 6.2 nV/deg^2^ for controls, mean R1 mfPhNR RAD value equal to 21.8 nV/deg^2^, and SD = 5.9 nV/deg^2^ for MS-ON group, we obtained 14 observations in both groups with α = 0.01 and a power = 0.90.

The differences of mfPhNR RAD mean values from each area (from Ring 1 to Ring 5, from sectors S1, ST, SN, IT, IN, and from the areas of the ETDRS sector analysis) between controls and MS-ON groups were analyzed by the one-way analysis of variance (ANOVA). Linear and exponential fittings were applied to describe the progression of the mfPhNR RAD values across rings (from R1 to R5), sectors (from ST to IT in the 5° to 20°) and ETDRS sectors (from T to I sectors in both 5° to 10° and 10° to 20°). *p*-Values lower than 0.01 was considered as statistically significant.

Minitab 17 (version 1) software was used for statistical analysis.

## 3. Results

We presented the mfPhNR of in Control and MS-ON eyes, following the three proposed topographies (see above) and the relative differences of RAD values in localized retinal areas between groups.

### 3.1. Ring Analysis

**Figure 2:**
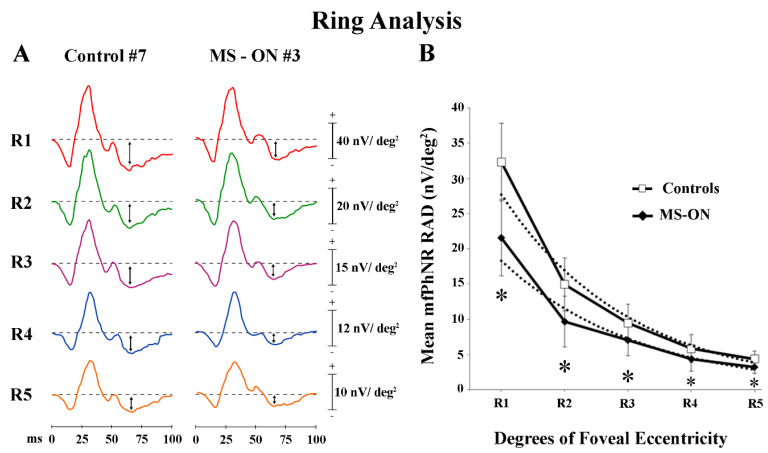
Multifocal photopic negative response (mfPhNR) ring (R) analysis. (**A**) MfPhNR averaged responses from ring 1 to 5 (R1 to R5) are presented from a representative control eye (#7) and from an eye of a multiple sclerosis patient affected by optic neuritis (MS-ON) (#3). The mean mfPhNR response amplitude density (RAD) measure is indicated by an arrow (**↕**). (**B**) Mean values of mfPhNR RAD (expressed in nanoVolt/degrees^2^, nV/deg^2^) plotted as a function of foveal eccentricities (R1 to R5 refer to ring analysis (see Methods)). Vertical bars represent one standard deviation of the mean values. Dashed lines indicate the exponential fitting for mfPhNR RADs (Controls: r^2^ = 0.97; MS-ON eyes: r^2^ = 0.95). The relative functions show a progressive decrease of mfPhNR RADs in both controls and MS-ON eyes with increasing eccentricities (from R1 to R5). The slope of the mean mfPhNR RAD function of both groups presents a greater steepness proceeding from R2 to R3 (the center to periphery transitional retinal areas: from 5° to 10° to 10° to 15° of foveal eccentricity). * indicates the statistically significant (*p* < 0.01) difference between MS-ON and control groups. The values of the statistical analysis are reported in Table 1.

In Figure 2A, mfPhNR traces from one representative control (#7) and one MS-ON (#3) eye are reported for the five rings with increasing eccentricity from the fovea (from 0° to 25°).

As reported in Table 1, in control eyes, the mean mfPhNR RAD values were found higher in the central rings (R1) and lower values in the more peripheral rings (R4 and R5). A similar difference of mfPhNR RAD values between rings was found in MS-ON eyes. However, the mean mfPhNR RAD values detected in MS-ON eyes were significantly (*p* < 0.01) reduced as compared to control ones for all rings (R1–R5).

When mean values of mfPhNR RAD recorded in controls and in MS-ON eyes were plotted as a function of retinal eccentricity (0°–25°), a progressive decrease of RAD values was observed from R1 to R5 in both groups (exponential fitting: controls: r^2^ = 0.97; MS-ON: r^2^ = 0.95). The same shape of the curves was observed in both groups, however, with lower values in MS-ON eyes (see Figure 2B). Moreover, in both groups, the slope of the function describing the progression of mean mfPhNR RAD showed a different steepness when proceeding from R1 to R2 as compared from R3 to R5. This difference corresponds to the center-to-peripheral transitional area (center to periphery: R2, 5° to 10° of foveal eccentricity).

As reported in Table 2, in both groups, when comparing mfPhNR RAD values from all rings and each ring versus all other rings separately, we found statistically significant differences (*p* < 0.01).

### 3.2. Sector Analysis

**Figure 3:**
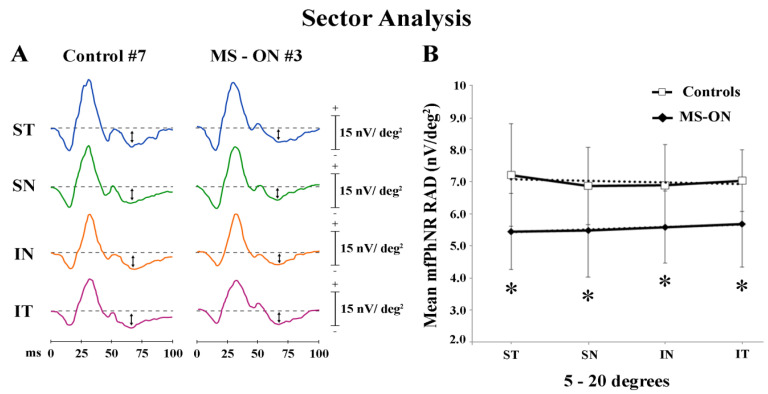
Multifocal photopic negative response (mfPhNR) sector analysis. (**A**) MfPhNR averaged are presented from a representative control eye (#7) and from an eye of a multiple sclerosis patient affected by optic neuritis (MS-ON) (#3). The mean mfPhNR response amplitude density (RAD) measure is indicated by an arrow (**↕**). We analyzed five sectors covering an area of 20° of eccentricity from the fovea. The first sector (S1) corresponding to R1 (0–5°) is reported on Figure 1; the more external sectors (between 5° to 20°) were quarters of annulus, localized in the superior-temporal (ST), superior-nasal (SN), inferior-nasal (IN), and inferior-temporal (IT) areas with respect to the fovea. (**B**) Mean values of mfPhNR RAD (expressed in nanoVolt/degrees^2^, nV/deg^2^) are plotted as a function sector analysis. Vertical bars represent one standard deviation of the mean values. Dashed lines indicate the linear fitting for mfPhNR RADs (controls: r^2^ = 0.17, MS-ON: r^2^ = 0.96). The relative functions show almost constant values of mfPhNR RADs in both controls and MS-ON eyes between sectors although the trend is actually linear only in the MS-ON group. * indicates the statistically significant (*p* < 0.01) difference between MS-ON and control groups. The values of the statistical analysis are reported in Table 3.

In Figure 3A, mfPhNR traces from one representative control (#7) and one MS-ON (#3) eye are reported for the four sectors covering an area between 5° to 20° of eccentricity from the fovea.

In the sector analysis, the central area corresponds with R1 described in *Ring analysis* results. As reported in Table 3, when considering the four sectors localized in the 5°–20° (ST, SN, IN, and IT) of eccentricity, we found that the mean values of mfPhNR RAD from MS-ON eyes were significantly (*p* < 0.01) reduced in all sectors as compared to control eyes.

When mean values of mfPhNR RAD from four sectors in the 5°–20°, recorded in controls and in MS-ON eyes, were plotted as a function of retinal eccentricity, we observed that RAD values in MS-ON eyes were different from those of control eyes; however, in both groups, mean mfPhNR responses obtained from ST, SN, IN, and IT showed almost constant values but different trends (linear fitting: controls: r^2^ = 0.17; MS-ON: r^2^ = 0.96) (see Figure 3B).

Moreover, as described in Table 4, when comparing the mean values of mfPhNR RAD derived from each singular sector with the others, no significant (*p* > 0.01) differences were observed in both groups.

### 3.3. ETDRS Sector Analysis

**Figure 4:**
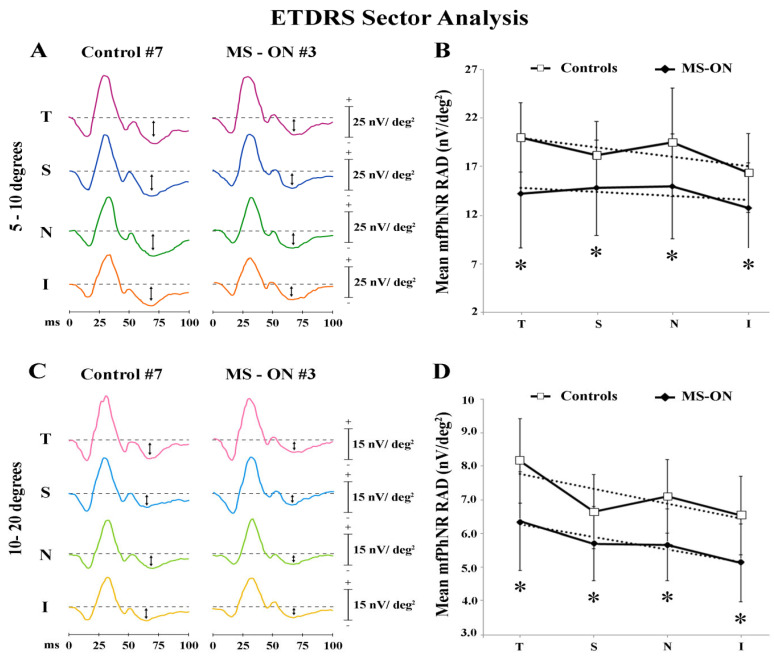
Multifocal photopic negative response (mfPhNR) ETDRS sector analysis. MfPhNR averaged are presented from a representative control eye (#7) and from an eye of a multiple sclerosis patient affected by optic neuritis (MS-ON) (#3). The mean mfPhNR response amplitude density (RAD) measure is indicated by an arrow (**↕**). We analyzed nine sectors covering an area of 20° of eccentricity from the fovea. The first sector (S1) corresponding to R1 (0–5°) is reported on Figure 1; the more external sectors between 5° to 10° (**A**) and between 10° to 20° (**C**) were quarters of annulus, localized in the temporal (T), superior (S), nasal (N), and inferior (I) areas with respect to the fovea. Mean values of mfPhNR RAD (expressed in nanoVolt/degrees^2^, nV/deg^2^) are plotted as a function of sector analysis on 5° to 10° (**B**) and between 10° to 20° (**D**). Vertical bars represent one standard deviation of the mean values. Dashed lines indicate the linear fitting for mfPhNR RADs (5°–10°: controls: r^2^ = 0.58, MS-ON: r^2^ = 0.28; 10°–20°: Controls: r^2^ = 0.59, MS-ON: r^2^ = 0.91). The relative functions show almost constant values of mfPhNR RADs in both controls and MS-ON eyes between sectors. * indicates the statistically significant (*p* < 0.01) difference between MS-ON and control groups. The values of the statistical analysis are reported in Table 5.

In Figure 4A,C, mfPhNR traces from one representative control (#7) and one MS-ON (#3) eye are reported for the five ETDRS sectors covering an area of 5°–10° of foveal eccentricity and for the four outermost ETDRS sectors covering an area of 10°–20° of foveal eccentricity, respectively.

When considering the ETDRS sector analysis configuration, the central area (R1) corresponds with R1 described in *Ring analysis* results. As reported in Table 5, mean mfPhNR RAD values were slightly higher in the T and in the N sectors both within 5°–10° and 10°–20° in control eyes, whereas more homogeneous mean mfPhNR RADs were found within 5°–10° and within 10°–20° in MS-ON eyes. Moreover, the mean values of mfPhNR RAD recorded in MS-ON eyes and derived from all localized ETDRS sectors (both from 5°–10° and from 10°–20°) were significantly (*p* < 0.01) reduced as compared to control eyes.

Moreover, when mean values of mfPhNR RAD from ETDRS sectors, recorded in controls and in MS-ON eyes, were plotted as a function of retinal eccentricities, we observed that values from MS-ON eyes differed from those of control eyes. However, in both groups mean mfPhNR RADs obtained from ETDRS sectors showed almost constant values at both 5°–10° (see Figure 4B) and 10°–20° (see Figure 4D) retinal eccentricities (ETDRS 5°–10° linear fitting: controls: r^2^ = 0.58 and in MS-ON: r^2^ = 0.28; ETDRS 10°–20° linear fittings in controls: r^2^ = 0.59 and in MS-ON: r^2^ = 0.91).

Table 6 reports the statistical analysis of differences between mfPhNR RADs from each ETDRS sector in both groups at both retinal eccentricities (5°–10° and 10°–20°). In control eyes, we found a significant (*p* < 0.01) difference between mean values of mfPhNR RAD exclusively comparing T and I sectors within 5°–10° and within 10°–20° and comparing T and N sectors within 10°–20°, whereas in MS-ON eyes, when comparing the mean values of mfPhNR RAD between ETDRS sectors, we found that only the comparison between T and I sectors within 10°–20° was significantly (*p* < 0.01) different. In both groups, the mfPhNR RAD values observed in each ETDRS sectors within 10°–20° were significantly (*p* < 0.01) reduced as compared to the corresponding sectors within 5°–10°.

## 4. Discussion

In the present pivotal study, we aimed to assess the function of the IRL and specifically of the RGCs located in selected retinal areas by using mfPhNR recordings both in control eyes and in eyes with documented morphological impairment of RGCs and concomitant absence of morpho-functional changes of ORL by considering the retrobulbar optic neuritis in multiple sclerosis as a model of neurodegeneration.

Indeed, for this purpose, we set out to verify whether the mfPhNR paradigm may be potentially considered as an indicator of localized RGCs dysfunction.

As a novel approach, we evaluated the mfPhNR data in localized retinal areas by using three retinal topographies in both groups, such as:

(1) The ring analysis, based on what is commonly studied in the mfERG responses in retinal pathologies to differentiate the function of ORL elements located in central and in peripheral retinal areas;

(2) The sector analysis, based on what has been previously described in the early application of mfPhNR in the glaucomatous optic neuropathy [32,39] with a specific study of the inferior-superior or the nasal-temporal changes of mfPhNR responses in five retinal sectors enclosed within 20° of foveal eccentricity;

(3) The ETDRS sector analysis, based on the potential correlation of mfPhNR data with morphological outcomes on the density of RGCs at the posterior pole by applying the common Sd-OCT segmentation analysis [46,53].

Data obtained by the three different analyses are separately discussed.

### 4.1. MfPhNR Findings by Applying Ring Analysis

The ring analysis is the usual electrophysiological configuration for investigating on the variability of the activity of the retinal neurons as a function of increasing foveal eccentricity. This is based on the retinal anatomy and on the neurophysiology of the neural components of the visual system in normal eyes. Indeed, because of the decreasing ganglion cell/receptor ratio, RGCs have a packing density, which is the highest in the perifoveal area and has a steeper drop-off with eccentricity, whereas the receptive field sizes increase with distance from the fovea [54,55].

In details, it has been clarified that PhNR signal originates from inner retinal cells and specifically from RGCs [27,28,29]. Therefore, from the available literature, simplistically, it might be expected that because the amplitude of the PhNR maps on to the distribution of RGCs and because PhNR amplitude increases with increased stimulus area, PhNR amplitude would increase linearly with increasing RGCs count. However, this is not the case since it has been reported that PhNR amplitude variations could be influenced by non-RGC neurons and by eccentricity-related changes in RGCs [56].

Since the absolute mfPhNR amplitude itself is mainly influenced by RGCs density and by stimulus size area [34], we believed that in the analysis of RGCs function, it was more reliable to evaluate the ratio between the bioelectrical response and the retinal area from which the responses were derived.

In this way, as for mfERG [19,20,21,22,23,24,25,26], we considered for the mfPhNR data the measure of amplitude/area that is the response amplitude density (RAD). In the arrangement of ring analysis, this choice was made to investigate the mfPhNR changes with increasing stimulus area from the foveal center up to the periphery (25°).

Firstly, it is interesting that in control eyes, we found higher mean mfPhNR RAD values in the central rings (R1 and R2) as compared to the remaining peripheral annular rings with increasing eccentricity from the fovea (R3 to R5), as reported in Figure 2B and in Table 1. In addition, when comparing mean mfPhNR data from the central rings moving towards the peripheral rings, we found that this difference was highly statistically significant (Table 2), meaning that RGCs function of the foveal center is much higher than in the peripheral areas.

Taken together, these findings suggest that in normal eyes, by using a functional measure of response density, such as RAD, it is possible to establish that a centro-peripheral difference of RGCs function (measured by mfPhNR RAD) exists, with this being higher in the foveal area (0°–5°) and decreasing sharply from the parafoveal area (5°–10°) up to the periphery (25°). These topographical functional differences are consistent with all that previously reported on the fact that RGCs numerical density decreases when receptive field size increases with eccentricity from the fovea [39,54,57].

When we analyzed data from MS-ON eyes, we found a significant reduction of mfPhNR RADs in all rings as compared to controls. Like controls, a trend to a reduction of mean mfPhNR RAD from the central retinal areas to the peripheral ones (Table 1 and Table 2) was found.

We considered our MS-ON eyes as a selective model of RGCs neurodegeneration since in the studied patients, a normal functional condition of pre-ganglionic elements detected by normal mfERG recordings and normal ORL morphology [26] were found.

It may be hypothesized that in presence of post-neuritis neurodegenerative process, the RGCs function of the central retinal areas is more impaired than that of the peripheral ones.

Our results suggest that mfPhNR recordings can be applied to several other pathologies characterized by localized RGCs impairment, such as retinal and post-retinal diseases presenting, for example, with concentric constriction and/or central scotoma of the visual field.

### 4.2. MfPhNR Findings by Applying Sector Analysis

In control eyes, when considering the sector analysis configuration, no statistically significant differences of mean mfPhNR RADs between each examined sector (ST, SN, IN, and IT) within 5°–20° of eccentricity (Table 3) were found.

This led us to believe that the RGCs function assessed by mfPhNR RAD should be similar in the analyzed sectors in normal condition. Contrary to the available histological data on RGCs density, describing a higher representation of these element in the nasal retina as compared to the distribution in different other sectors [54], we did not find functional changes of RCGs among the examined sectors in normal eyes.

In MS-ON eyes, statistically significant reduced mfPhNR RAD values were found when compared to controls in each examined sector. This suggests that by examining quarters of retinal areas, it is possible to record the selected RGCs function from normal eyes and to establish a difference from that of MS-ON eyes, which are expected and confirmed to be impaired.

Contrary to all that is observed in glaucomatous patients in which the RGCs dysfunction (reduced mfPhNR responses) was different between sectors [39], no significant differences were found between sectors. This indicates that in our model of neurodegeneration (MS-ON), the detected sector impairment should be not selective.

In our case, the linear fitting of mfPhNR RAD values recorded within 5° to 20° of foveal eccentricity from different sectors (Figure 3) in both groups confirmed that also analyzing the localized retinal areas by sectors, this electrophysiological paradigm was able to differentiate responses significantly between control and MS-ON eyes, being reduced in the latter group.

All that has been described could be of interest for the potential application of this methodology to pathologies that can affect differentially the function of RGCs located in different retinal sectors, such as glaucomatous optic neuropathy commonly showing different patterns of visual field impairment, ischemic optic neuropathy in the event of altitudinal scotoma, and post-retinal abnormalities involving the RGCs by retrograde degeneration, presenting with quadrantanopia or hemianopia.

### 4.3. MfPhNR Findings by Applying ETDRS Sector Analysis

The ETDRS map is the usual representation of morphological data regarding the retina and specifically of the retinal segmented data by means of Sd-OCT arrangement [46,53].

Based on our results, when considering mean mfPhNR RAD values recorded from the paracentral (5°–10°) and the peripheral (10°–20°) ETDRS areas, we found a statistically significant reduction in MS-ON eyes as compared to control eyes. This indicates that in the chosen neurodegenerative model of ON, it is possible to detect a functional impairment of RGCs in the examined ETDRS sectors.

In controls, when considering the comparisons among ETDRS sectors, we found a consistently higher RGCs function recorded from the T and the N sectors within both retinal eccentricities. In addition, when comparing mfPhNR data from each ETDRS sector, we found significantly different values between T and I sectors at 5°–10° and 10°–20° of eccentricity. Thus, we might suggest that the RGCs located in the ETDRS T sectors are functionally different from those located in the I sectors. This difference was significant also when comparing data between T and N sectors within 10°–20° of eccentricity.

Our functional findings cannot be compared to the few available data on the distribution of RGCs in normal eyes following the ETDRS configuration [58] based on the differently considered sample size.

In MS-ON eyes, a significant reduction of mfPhNR RAD values with respect to those of controls was found in all examined ETDRS sectors. Moreover, in MS-ON group, we found a statistically significant difference only between T and I sectors within 10°–20° of eccentricity. This could be ascribed to the possibility that the RGCs located in the ETDRS inferior sector (10°–20°) might be more involved in the degenerative process of ON.

In addition, in both groups, mfPhNR RAD values detected in ETDRS sectors within 10°–20° were significantly reduced when compared to that of 5°–10° of eccentricities. This means that also by applying the ETDRS configuration, it is possible to assess a significant decrease of RGCs function in more peripheral areas with respect to the fovea. Thus, as said above for the ring analysis, the ETDRS configuration reproduces the centro-peripheral difference of mfPhNR responses.

Our findings have potential remarkable implications in the clinical settings, considering the possibility to evaluate the electrofunctional (mfPhNR RAD) and the morphological (IRL segmented thickness or volume) RGCs topography for almost the same localized areas in several diseases affecting primarily or secondarily these retinal neurons.

It is important to underline that, in our study, we were not able to establish a correlation between RGCs functional and morphological data among ETDRS sectors in MS-ON eyes. This is because our cohort of MS-ON patients was selected based on similar values of inner macular volumes and thickness (thus not representing the common spread of Sd-OCT values in the ON event).

## 5. Conclusions

In conclusion, data obtained from the present pivotal study suggest that the mfPhNR recordings allow to detect a localized RGCs dysfunction in the pathologic condition in which a selective RGCs morphological involvement and a concomitant absence of morpho-functional changes of ORL may occur (MS-ON patients with fully recovered high-contrast BCVA).

In our human model of retinal neurodegeneration, this impairment can be observed independently from the methods of analysis used: ring analysis, sector analysis, and ETRDS sector analysis.

As a consequence, the mfPhNR should be taken in consideration as an electrophysiological paradigm for assessing localized RGCs dysfunction in other several pathologies (i.e., glaucomatous optic neuropathy, diabetes, ischemic optic neuropathy) in which potential localized RGCs impairment may lead to corresponding visual field defects or selective loss of RGCs and of retinal nerve fiber layer.

## Figures and Tables

**Table 1:** Mean values of multifocal photopic negative response (mfPhNR) ring analysis observed in Controls (C) compared to multiple sclerosis patients with optic neuritis (MS-ON).

		Ring 1 0–5 Degrees	Ring 2 5–10 Degrees	Ring 3 10–15 Degrees	Ring 4 15–20 Degrees	Ring 5 20–25 Degrees
		**RAD ^a^**	**RAD ^a^**	**RAD ^a^**	**RAD ^a^**	**RAD ^a^**
**Controls**	Mean	32.252	14.928	9.448	5.840	4.372
**N ^b^ = 25**	SD ^c^	5.487	3.734	2.698	1.941	1.120
**MS-ON**	Mean	21.515	9.63	7.02	4.31	3.19
**N ^b^ = 20**	SD ^c^	5.402	3.587	2.233	1.694	0.865
***A ^d^* vs. *C***	*f (1, 43)*	43.13	23.16	10.45	7.72	15.06
	*p*	0.000	0.000	0.002	0.008	0.000

^a^ RAD, response amplitude density (measured in nanoV/degree^2^); ^b^ N, number of eyes of each group; ^c^ SD, one standard deviation of the mean; ^d^ A, one-way analysis of variance. *p*-values < 0.01 were considered as statistically significant for group comparisons.

**Table 2:** Statistical differences between the multifocal photopic negative response (mfPhNR) ring analysis mean data obtained in different localized retinal areas in control eyes and in multiple sclerosis patients with optic neuritis (MS-ON).

	Ring 2 5–10 Degrees		Ring 3 10–15 Degrees		Ring 4 15–20 Degrees		Ring 5 20–25 Degrees	
**Controls**	***A ^a^ vs***		***A ^a^ vs***		***A ^a^ vs***		***A ^a^ vs***	
	*f(1, 48)*	*p*	*f(1, 48)*	*p*	*f(1, 49)*	*p*	*f(1, 48)*	*p*
**Ring 1**	170.33	0.000	346.52	0.000	514.83	0.000	619.62	0.000
**Ring 2**	---------		35.38	0.000	116.59	0.000	183.31	0.000
**Ring 3**	---------		---------		18.70	0.000	51.16	0.000
**Ring 4**	---------		---------		---------		10.83	0.002
**MS-ON**	***A ^a^ vs***		***A ^a^ vs***		***A ^a^ vs***		***A ^a^ vs***	
	*f(1, 38)*	*p*	*f(1, 38)*	*p*	*f(1, 38)*	*p*	*f(1, 38)*	*p*
**Ring 1**	67.19	0.000	122.98	0.000	184.71	0.000	224.40	0.000
**Ring 2**	---------		7.63	0.009	5.97	0.000	60.92	0.000
**Ring 3**	---------		---------		29.46	0.000	75.48	0.000
**Ring 4**	---------		---------		---------		10.73	0.002

^a^ A, one-way analysis of variance. *p*-values < 0.01 were considered as statistically significant for comparisons.

**Table 3:** Mean values of multifocal photopic negative response (mfPhNR) sector analysis between 5 to 20 degrees observed in controls (C) compared to multiple sclerosis patients with optic neuritis (MS-ON) ones.

		Ring 1 0–5 Degrees	Superior-Temporal 5–20 Degrees	Superior-Nasal 5–20 Degrees	Inferior-Nasal 5–20 Degrees	Inferior-Temporal 5–20 Degrees
		**RAD ^a^**	**RAD ^a^**	**RAD ^a^**	**RAD ^a^**	**RAD ^a^**
**Controls**	Mean	32.252	7.208	6.868	6.888	7.036
**N ^b^ = 25**	SD ^c^	5.487	1.598	1.211	1.270	0.962
**MS-ON**	Mean	21.515	5.448	5.481	5.586	5.676
**N ^b^ b = 20**	SD ^c^	5.402	1.190	1.458	1.128	1.342
***A ^d^* vs. *C***	*f (1, 43)*	43.13	16.78	12.16	12.88	15.66
	*p*	0.000	0.000	0.001	0.000	0.000

^a^ RAD, response amplitude density (measured in nanoV/degree^2^); ^b^ N, number of eyes of each group; ^c^ SD, one standard deviation of the mean; ^d^ A, one-way analysis of variance. *p*-values < 0.01 were considered as statistically significant for group comparisons.

**Table 4:** Statistical differences between different localized retinal areas of multifocal photopic negative response (mfPhNR) sector analysis data obtained between 5 to 20 degrees in control eyes and in multiple sclerosis patients with optic neuritis (MS-ON).

	Superior-Nasal 5–20 Degrees		Inferior-Nasal 5–20 Degrees		Inferior-Temporal 5–20 Degrees	
**Controls**	***A ^a^ vs***		***A ^a^ vs***		***A ^a^ vs***	
	*f(1, 48)*	*p*	*f(1, 48)*	*p*	*f(1, 48)*	*p*
**Superior-Temporal**	0.72	0.401	0.21	0.647	0.61	0.437
**Superior-Nasal**	---------		0.29	0.590	0.00	0.955
**Inferior-Nasal**	---------		---------		0.22	0.644
**MS-ON**	***A ^a^ vs***		***A ^a^ vs***		***A ^a^ vs***	
	*f(1, 38)*	*p*	*f(1, 38)*	*p*	*f(1, 38)*	*p*
**Superior-Temporal**	0.01	0.938	0.32	0.573	0.14	0.709
**Superior-Nasal**	---------		1.34	0.709	0.06	0.800
**Inferior-Nasal**	---------		---------		0.05	0.820

^a^ A, one-way analysis of variance. *p*-values < 0.01 were considered as statistically significant for comparisons.

**Table 5:** Mean values of multifocal photopic negative response (mfPhNR) sector analysis between 5 to 10 degrees and between 10 and 20 degrees observed in controls (C) compared to multiple sclerosis patients with optic neuritis (MS-ON) ones.

		**Ring 1** **0–5 Degrees**	**Temporal** **5–10 Degrees**	**Superior** **5–10 Degrees**	**Nasal** **5–10 Degrees**	**Inferior** **5–10 Degrees**
		**RAD ^a^**	**RAD ^a^**	**RAD ^a^**	**RAD ^a^**	**RAD ^a^**
**Controls**	Mean	32.252	19.972	18.144	19.468	16.328
**N ^b^ = 25**	SD ^c^	5.487	3.568	3.470	5.565	4.038
**MS-ON**	Mean	21.515	14.195	14.805	14.962	12.752
**N ^b^ = 20**	SD ^c^	5.402	5.546	4.888	5.365	4.614
***A ^d^* vs. *C***	*f (1, 43)*	43.13	17.92	7.17	7.85	7.68
	*p*	0.000	0.000	0.010	0.008	0.008
		**Ring 1:** **0–5 Degrees**	**Temporal** **10–20 Degrees**	**Superior** **10–20 Degrees**	**Nasal** **10–20 Degrees**	**Inferior** **10–20 Degrees**
		**RAD ^a^**	**RAD ^a^**	**RAD ^a^**	**RAD ^a^**	**RAD ^a^**
**Controls**	Mean	32.252	8.156	6.644	7.096	6.528
**N ^b^ = 25**	SD ^c^	5.487	1.467	1.104	1.064	1.161
**MS-ON**	Mean	21.515	6.357	5.690	5.657	5.119
**N ^b^ = 20**	SD ^c^	5.402	1.260	1.099	1.099	1.171
***A ^d^* vs. *C***	*f (1, 43)*	43.13	18.9	8.33	19.74	16.24
	*p*	0.000	0.000	0.010	0.008	0.008

^a^ RAD, response amplitude density (measured in nanoV/degree^2^); ^b^ N, number of eyes of each group; ^c^ SD, one standard deviation of the mean; ^d^ A, one-way analysis of variance. *p*-values < 0.01 were considered as statistically significant for group comparisons.

**Table 6:** Statistical differences between different localized retinal areas of multifocal photopic negative response (mfPhNR) sector analysis data obtained between 5 to 10 degrees and between 10 and 20 degrees in control eyes and in multiple sclerosis patients with optic neuritis (MS-ON).

	**Temporal** **5–10 Degrees**		**Inferior** **5–10 Degrees**		**Nasal** **5–10 Degrees**			
**Controls**	***A ^a^ vs***		***A ^a^ vs***		***A ^a^ vs***			
	*f(1, 48)*	*p*	*f(1, 48)*	*p*	*f(1, 48)*	*p*		
**Superior**	3.37	0.072	2.91	0.095	1.02	0.318		
**Temporal**	---------		11.43	0.001	3.37	0.072		
**Inferior**	---------		---------		5.21	0.027		
**MS-ON**	***A ^a^ vs***		***A ^a^ vs***		***A ^a^ vs***			
	*f(1, 38)*	*p*	*f(1, 38)*	*p*	*f(1, 38)*	*p*		
**Superior**	0.14	0.714	1.87	0.180	0.01	0.923		
**Temporal**	---------		0.80	0.377	0.20	0.659		
**Inferior**	---------		---------		1.95	0.171		
	**Temporal** **10–20 Degrees**		**Inferior** **10–20 Degrees**		**Nasal** **10–20 Degrees**			
**Controls**	***A ^a^ vs***		***A ^a^ vs***		***A ^a^ vs***		***A ^a^* vs. *5–10 degrees***	
	*f(1, 48)*	*p*	*f(1,48)*	*p*	*f(1, 48)*	*p*	*f(1, 48)*	*p*
**Superior**	16.95	0.000	0.13	0.719	3.25	0.078	249.35	0.000
**Temporal**	---------		18.93	0.000	8.55	0.005	234.53	0.000
**Inferior**	---------		---------		3.25	0.078	136.01	0.000
**Nasal**	---------		---------		---------		119.21	0.000
**MS-ON**	***A ^a^ vs***		***A ^a^ vs***		***A ^a^ vs***		***A ^a^* vs. *5–10 degrees***	
	*f(1, 38)*	*p*	*f(1, 38)*	*p*	*f(1, 38)*	*p*	*f(1, 38)*	*p*
**Superior**	3.18	0.082	2.53	0.120	0.01	0.925	66.20	0.000
**Temporal**	---------		10.36	0.003	3.51	0.069	37.99	0.000
Inferior	---------		---------		2.24	0.142	51.42	0.000
Nasal	---------		---------		---------		57.74	0.000

^a^ A, one-way analysis of variance. *p*-values < 0.01 were considered as statistically significant for comparisons.

## Data Availability

Data available from authors.

## Abbreviations

MS	multiple sclerosis
ON	optic neuritis
MS-ON	multiple sclerosis patients with optic neuritis followed by good recovery of best corrected visual acuity
Ff-ERG	Full-field Electroretinogram
PhNR	Photopic Negative Response
Ff-PhNR	Full-field Photopic Negative Response
PERG	pattern electroretinogram
MfERG	multifocal electroretinogram
Mf-PhNR	multifocal Photopic Negative Response
RGCs	retinal ganglion cells
BCVA	best corrected visual acuity
RAD	response amplitude density
OCT	optical coherence tomography
IRL	innermost retinal layers
ORL	outer retinal layers
SD	one standard deviation of the mean
N	number of eyes of each group
A	one-way analysis of variance
R	ring
ST	superior-temporal
SN	superior-nasal
IN	inferior-nasal
IT	inferior-temporal
S	superior
T	temporal
I	inferior
N	nasal
I-MV	IRL macular volume
I-MT	IRL macular thickness
ETDRS	Early Treatment of Diabetic Retinopathy Study
